# Social Vulnerability and Groundwater Vulnerability to Contamination From Unconventional Hydrocarbon Extraction in the Appalachian Basin

**DOI:** 10.1029/2022GH000758

**Published:** 2023-04-13

**Authors:** Mario A. Soriano, Joshua L. Warren, Cassandra J. Clark, Nicholaus P. Johnson, Helen G. Siegel, Nicole C. Deziel, James E. Saiers

**Affiliations:** ^1^ School of the Environment Yale University New Haven CT USA; ^2^ Integrated GroundWater Modeling Center High Meadows Environmental Institute Princeton University Princeton NJ USA; ^3^ Department of Biostatistics School of Public Health Yale University New Haven CT USA; ^4^ Department of Environmental Health Sciences School of Public Health Yale University New Haven CT USA

**Keywords:** environmental justice, groundwater vulnerability, hydraulic fracturing, risk assessment, unconventional oil and gas

## Abstract

Unconventional oil and gas (UOG) development, made possible by horizontal drilling and high‐volume hydraulic fracturing, has been fraught with controversy since the industry's rapid expansion in the early 2000's. Concerns about environmental contamination and public health risks persist in many rural communities that depend on groundwater resources for drinking and other daily needs. Spatial disparities in UOG risks can pose distributive environmental injustice if such risks are disproportionately borne by marginalized communities. In this paper, we analyzed groundwater vulnerability to contamination from UOG as a physically based measure of risk in conjunction with census tract level sociodemographic characteristics describing social vulnerability in the northern Appalachian Basin. We found significant associations between elevated groundwater vulnerability and lower population density, consistent with UOG development occurring in less densely populated rural areas. We also found associations between elevated groundwater vulnerability and lower income, higher proportions of elderly populations, and higher proportion of mobile homes, suggesting a disproportionate risk burden on these socially vulnerable groups. We did not find a statistically significant association between elevated groundwater vulnerability and populations of racial/ethnic minorities in our study region. Household surveys provided empirical support for a relationship between sociodemographic characteristics and capacity to assess and mitigate exposures to potentially contaminated water. Further research is needed to probe if the observed disparities translate to differences in chemical exposure and adverse health outcomes.

## Key Points

- Groundwater vulnerability to contamination from unconventional oil and gas development was assessed using a physically based modeling framework
- Elevated groundwater vulnerability was associated with several sociodemographic indicators of high social vulnerability
- Co‐occurrence of groundwater vulnerability and social vulnerability poses distributive environmental injustice

## 1 Introduction

Unconventional oil and gas (UOG) development refers to the extraction of hydrocarbons from shale and other low permeability formations using a combination of horizontal drilling and high‐volume hydraulic fracturing. Hydraulic fracturing (“fracking”) involves the injection of millions of gallons of water mixed with sand and chemicals at high pressures to induce openings in the target formation and ease the flow of trapped oil and gas. UOG development has been hailed for its socioeconomic benefits, allowing the United States of America (USA) to become a net energy exporter in recent years and facilitating the transition away from coal toward lower carbon dioxide‐emitting natural gas (Mayfield et al., 2019; Tanaka et al., 2019). With natural gas viewed as a bridge fuel toward renewable energy sources, the UOG industry is projected to continue to expand domestically and globally in the coming decades (EIA, 2021). The ongoing pandemic‐ and conflict‐induced global disruption in energy supplies is bolstering the USA's continued expansion of its UOG industry and export capacity for its international allies (Zakeri et al., 2022).

Despite the economic and geopolitical significance of UOG development, concerns over risks posed by the water‐intensive industry on the environment and public health remain (Clark et al., 2021; Zhang et al., 2021). Such questions are particularly salient in many rural communities hosting intensive UOG development colocated with local populations depending on private water wells for their daily needs. Concerns of drinking water contamination are exacerbated by the toxicity of some chemical additives used in hydraulic fracturing fluids and wastewaters generated by the industry (Elliott et al., 2017; Wollin et al., 2020). Potential contamination pathways have been identified across all stages of UOG development and involve multiple mechanisms such as surface spills, leaking wellbores, and subsurface migration of contaminants (EPA, 2016; Li et al., 2021; Llewellyn et al., 2015; Siegel et al., 2022; Vengosh et al., 2014; Woda et al., 2018; Xiong et al., 2022). However, several studies have also suggested that drinking water contamination is highly localized and found no systematic patterns at regional scales (Barth‐Naftilan et al., 2018; Epuna et al., 2022; Wen et al., 2018, 2021). Nevertheless, an increasing number of epidemiological studies have suggested drinking water as a potential exposure route linking UOG development to adverse health effects, including pregnancy outcomes, respiratory symptoms, and cancer incidence, among others (Clark, Johnson, et al., 2022; Deziel et al., 2020; Hill & Ma, 2022; Wollin et al., 2020).

Examining social disparities in the spatial distribution of environmental risks (e.g., whether hazardous facilities are disproportionately located in marginalized neighborhoods) is the subject of environmental justice scholarship and reflects the concept of distributive justice. In the case of UOG, the empirical evidence to date has shown some mixed indications of disproportionate placement of risks—including drilling, siting of pipelines and wastewater disposal facilities, toxic emissions, water quality complaints, and negative health outcomes—on marginalized communities (Clark et al., 2021; Clough, 2018; Emanuel et al., 2021; Johnston et al., 2020; Kroepsch et al., 2019; Ogneva‐Himmelberger & Huang, 2015; Silva et al., 2018; Zwickl, 2019). Studies vary with respect to the type and description of environmental hazards, the considered measures of social disadvantage, and the statistical approaches implemented.

Previous research on distributive justice have used measures of proximity to UOG, including distance to nearest UOG well, number of UOG wells within predefined radial distances, and inverse distance weighted UOG well counts, as the primary proxy for risk (Clark, Xiong, et al., 2022; Kroepsch et al., 2019). These proximity‐based approaches capture aggregate risk but cannot identify specific risk mechanisms, for example, differentiating risks from air pollution versus drinking water pollution (Adgate et al., 2014; Deziel, Clark, et al., 2022). To better characterize groundwater‐specific risk pathways, recent studies applied the concept of groundwater vulnerability to contamination within the context of UOG. Understood through the lens of a source‐pathway‐receptor framework, groundwater vulnerability to contamination describes the likelihood for a contaminant released from a known source (e.g., a UOG well pad) to reach a specified receptor (e.g., a drinking water well) (Focazio et al., 2003; Wachniew et al., 2016). Groundwater vulnerability can be assessed using physically based modeling to simulate contaminant trajectories from UOG well pad locations, thereby providing a spatially explicit approach for quantifying the risks posed by UOG on drinking water resources (Mallants et al., 2022; Soriano et al., 2020, 2021). Recently, Soriano et al. (2022) modeled vulnerability in a 104,000‐sq km region in the northern Appalachian Basin and estimated that about 21,000–30,000 people were dependent on domestic groundwater wells that were vulnerable to contamination from UOG. The sociodemographic characteristics of the communities who bear this physical groundwater vulnerability and the potential implications for environmental injustice have not been explored.

In this paper, we evaluate spatial associations between physically based groundwater vulnerability to contamination and a broad selection of sociodemographic characteristics to examine potential environmental injustice related to the distribution of UOG risks. We examine relationships between estimates of regional groundwater vulnerability from Soriano et al. (2022) and social vulnerability metrics in the form of 15 census tract‐based sociodemographic variables (Flanagan et al., 2011). We supplement our regional scale analysis with household‐level data on socioeconomic characteristics and groundwater use patterns obtained from structured questionnaire surveys we conducted between 2019 and 2020 to examine if disparities in vulnerability are exacerbated by disparities in household capacities to mitigate exposures. By integrating a physics‐based measure of drinking water contamination risks with robust measures of social vulnerability grounded in local homeowner surveys, this paper demonstrates a novel and scientifically defensible analysis of distributive environmental justice in the context of UOG development.

## 2 Materials and Methods

### 2.1 Physically Based Groundwater Vulnerability to Contamination

**Figure 1:**
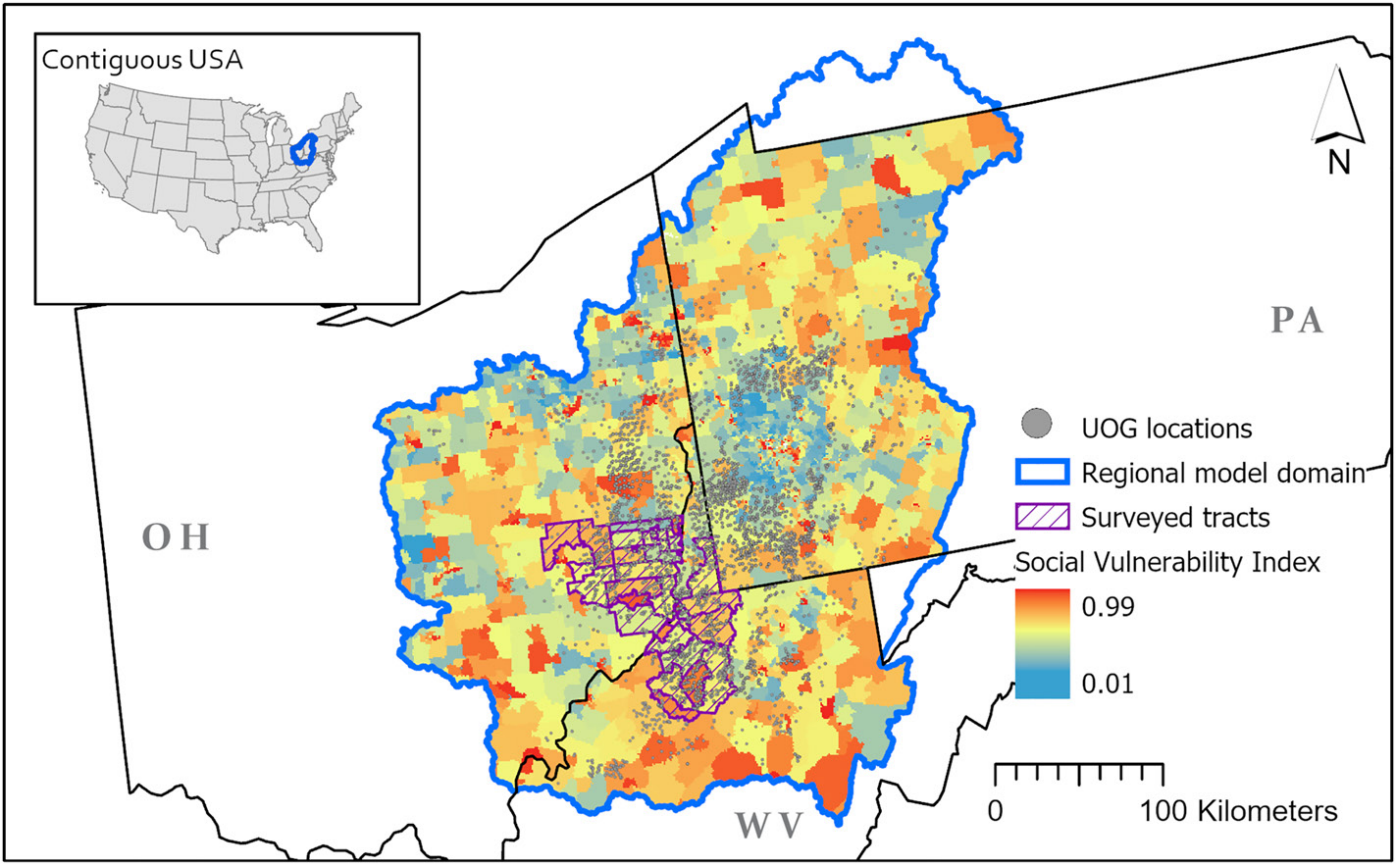
The physically based groundwater vulnerability model encompasses a 104,000‐sq km area in Pennsylvania, Ohio, and West Virginia (regional model domain). Colors show the CDC's Social Vulnerability Index, with higher values indicating greater social vulnerability. Hatch lines show census tracts where 210 homeowners were surveyed.

We evaluated groundwater vulnerability to contamination from UOG (GWV) for a 104,000‐sq km region (the regional model domain) in the Appalachian Basin, covering parts of Pennsylvania, Ohio, and West Virginia (Figure 1 and Figure S1 in Supporting Information S1). The region hosts intensive UOG development of the Marcellus and Utica shale formations. The Appalachian Basin has been identified as a hotspot for environmental injustice related to water, and these inequalities may be amplified by colocation with UOG operations (Mueller & Gasteyer, 2021). We quantified GWV using an ensemble framework that employed physically based groundwater flow modeling and forward particle tracking. We adapt a conceptual model of the surficial aquifer system from Zell and Sanford (2020a, 2020b), which represents unconfined aquifers in areas with unconsolidated deposits and weathered and fractured regolith in areas with consolidated deposits. We discretized the regional model domain into 250 m × 250 m grid cells and generated an ensemble of flow models calibrated using 11,766 groundwater level measurements. For every model in the ensemble, we simulated contamination events by releasing particles at UOG source locations (well pads) and tracking the pathways where the contaminants were transported by groundwater flow. The approach focuses on contaminant release mechanisms at well pads as these have been demonstrated to be the most likely pathway for environmental release of UOG contaminants and does not account for possible mechanisms away from well pads that have lower probabilities, such as roadside truck spills (Faber et al., 2019; Rozell & Reaven, 2012; Shanafield et al., 2019). GWV, defined as the likelihood for contaminants from UOG to reach specified locations in the domain, was calculated for each grid cell as the proportion of models in the ensemble where particle tracks intersected that grid cell. GWV ranges from 0 to 1, with higher values indicating a higher likelihood of adverse impact in the event that contaminants are released from UOG source locations. Note that groundwater vulnerability alone does not equate to contamination, as the latter is contingent on both vulnerability and actualized hazards, namely, the occurrence of contaminant releases from UOG sources. GWV is controlled by topography, hydrogeology (e.g., the spatial distribution of permeability), and the spatial distribution of potential contaminant sources. Full details of the physically based analysis are provided in Soriano et al. (2022).

### 2.2 Social Vulnerability

To assess social vulnerability, we analyzed the 15 sociodemographic characteristics comprising the Centers for Disease Control and Prevention's Social Vulnerability Index (SVI, Table 1 and Table S1 in Supporting Information S1) for census tracts inside the regional model domain (Flanagan et al., 2011). These characteristics are grouped into four themes: Socioeconomic Status; Household Composition & Disability; Minority Status & Language; and Housing Type & Transportation. The SVI data set has been previously used to elucidate disparities in the spatial distribution of environmental risks related to gas pipeline placement, recurring natural hazards, and negative health outcomes (Deziel, Warren, et al., 2022; Emanuel et al., 2021; Flanagan et al., 2018; Lehnert et al., 2020). Rather than using the composite SVI metric, we analyzed the underlying sociodemographic variables designated by the “EP_” prefix in the 2018 SVI release, which express population‐related characteristics as percentages of the total for each census tract based on the 2013–2018 American Community Survey (Centers for Disease Control and Prevention/Agency for Toxic Substances and Disease Registry/Geospatial Research Analysis and Services Program, 2020). We account for the total population and size of the census tracts by including population density in the analyses.

Because our analysis uses sociodemographic data from 2013 to 2018, we cannot make comparisons between scenarios of pre‐UOG development and contemporary UOG development in the region. Such longitudinal analysis can elucidate how UOG development contributes to socioeconomic and demographic changes, which is a question that warrants further study but one we do not address in this paper. Here, our focus is on the question of whether there are apparent inequities in the distribution of concurrent UOG contamination risks and societal outcomes across the population.

### 2.3 Statistical Analyses

We analyzed census tracts where the proportion of the population dependent on domestic groundwater supplies was ≥10% (*n* = 647) within the physically based model domain (Murray et al., 2021) (Figure S1 in Supporting Information S1). We focused on areas where people depend on private wells completed in shallow aquifers and excluded areas primarily served by public water systems because domestic wells are more likely to be impacted by surface spills, which are the most common mechanism for release of UOG contaminants to the environment (Patterson et al., 2017; Shanafield et al., 2019). We aggregated GWV from the model grid into tract level areal averages and categorized each census tract as having an elevated or not elevated level of GWV using a threshold value of 0.001. We selected this threshold based on the US EPA's upper limit of 10^−4^ for acceptable risks (Haas et al., 2014). We conceptualize risk as the product of vulnerability and hazard, where hazard corresponds to UOG spill probabilities ranging from 0.01 to 0.1 (Shanafield et al., 2019). Thus, vulnerability must be less than 0.001 to remain within the acceptable risk. We conducted a bivariate analysis using two‐sample Welch *t*‐tests to describe differences in individual SVI characteristics between census tracts grouped by their GWV category. We then performed multivariable logistic regression to evaluate the associations between the multiple SVI characteristics and GWV simultaneously using a hierarchical Bayesian spatial generalized linear mixed model. This multivariable logistic regression approach models the log odds of a census tract having elevated GWV as a function of the SVI characteristics and population density while accounting for spatial autocorrelation using spatially correlated random effects. Accounting for spatial correlation enables accurate statistical inference for the regression associations of interest. We modeled the random effects using a conditional autoregressive prior proposed by Leroux et al. (2000), which defines spatial correlation based on the geographic connections between census tracts. We specified standard weakly informative prior distributions for all model parameters (Table S2 in Supporting Information S1). Bayesian inference was made based on posterior samples collected from a Markov chain Monte Carlo sampling algorithm, where we removed 50,000 iterations during a burn‐in period prior to convergence. We further thinned the remaining 100,000 samples by a factor of 10 to reduce posterior autocorrelation, resulting in 10,000 samples for use in posterior inference. We tested statistical models specified using all 16 predictors (15 SVI characteristics + population density; “allvar” models) and models specified using subsets of the predictors selected via forward and backward stepwise regression (“varsel” models). Statistical models were compared using the Deviance Information Criterion (DIC) and corresponding effective number of parameters (p_D_), while convergence was assessed with the Geweke diagnostic and effective sample size for each parameter in the model (Geweke, 1991; Spiegelhalter et al., 2002). We further tested the sensitivity of our results by: (a) using an alternative threshold for elevated GWV of 0.01, and (b) retaining non‐urban census tracts whose centroids lie outside census urbanized areas (*n* = 646), in contrast to the original criterion based on the proportion of population dependent on domestic groundwater (U.S. Census Bureau, 2021b). All statistical analyses were performed in R v4.1.0, with packages CARBayes, rgdal, sf, and spdep (Bivand et al., 2022; Bivand et al., 2013; Lee, 2013; Pebesma, 2018; R Core Team, 2021).

### 2.4 Household Survey

We also conducted structured questionnaire surveys in 210 households distributed across 32 census tracts in Ohio and West Virginia over the period of May–August 2019 and October 2020 (Figure 1). This household‐level analysis serves to complement the census tract‐based vulnerability analyses described above by allowing us to probe household‐level factors that may exacerbate disparities in groundwater contamination risks. Participants were recruited by fliers, postcards, and social media ads targeted toward the study region. Interested participants were screened for eligibility based on the home being served by either a private groundwater well or spring, the survey respondent being an adult household decision‐maker (≥21 years old), and an ability to communicate in English. We anticipate that the language criterion introduced negligible bias to our sample due to the low prevalence of non‐English speaking populations in target communities (see Table 1). Survey questions include demographic and socioeconomic characteristics, as well as questions pertaining to residential characteristics and household water use patterns that capture household ability to mitigate drinking water exposures (e.g., consumption of bottled water, presence of water treatment systems). Trained interviewers administered the surveys in‐person in 2019 and over the telephone in 2020. Prior informed consent was obtained from all participants according to protocols approved by the Institutional Review Board of Yale University (HIC #2000021809) and the US Environmental Protection Agency (HSR‐001162).

**Table 1:** Sociodemographic Characteristics of Census Tracts Within the Regional Model Domain Categorized by Groundwater Vulnerability to Contamination

	Tracts with ≥10% gw dependent population (*n* = 647)		
Sociodemographic characteristic	GWV ≥ 0.001 (*n* = 362)	GWV < 0.001 (*n* = 285)	*p*‐value^a^
SVI Theme: Socioeconomic Status			
Below poverty, %	10.8 (7.3–15.1)	10.0 (5.6–14.2)	0.031
Unemployed, %	5.0 (3.5–6.6)	4.5 (2.8–6.0)	0.011
Income per capita, USD	27,230 (24,386–31,321)	28,532 (24,888–34,643)	<0.0001
No high school diploma, %	9.2 (6.7–12.9)	8.4 (5.5–11.7)	0.411
SVI Theme: Household Composition & Disability			
Aged 65 or older, %	20.2 (18.0–22.4)	19.4 (16.2–22.0)	0.002
Aged 17 or younger, %	19.9 (17.9–21.8)	21.0 (18.4–23.7)	<0.001
Civilian with a disability, %	15.9 (13.2–18.8)	14.5 (11.4–16.8)	<0.0001
Single‐parent households, %	6.2 (4.3–8.2)	6.2 (4.0–8.7)	0.374
SVI Theme: Minority Status & Language			
Minority^b^, %	3.0 (1.9–4.7)	4.2 (2.4–6.9)	0.002
Aged 5 or older who speaks English “less than well”, %	0.0 (0.0–0.3)	0.1 (0.0–0.5)	0.071
SVI Theme: Housing Type & Transportation			
Multi‐unit structures^c^, %	0.4 (0.0–2.1)	1.2 (0.0–3.9)	0.001
Mobile homes, %	12.9 (7.3–18.0)	7.1 (0.9–14.2)	<0.0001
Crowding^d^, %	0.8 (0.1–1.6)	0.7 (0.0–1.6)	0.899
No vehicle, %	4.4 (2.8–6.5)	4.6 (2.6–7.1)	0.176
Group quarters^e^, %	0.2 (0.0–1.6)	0.2 (0.0–1.9)	0.644
Population density, persons/sq mi	96.8 (50.6–178.1)	215.2 (102.0–650.9)	<0.0001

*Note*. Data are shown as medians (25th–75th percentiles). Detailed descriptions of the sociodemographic characteristics are given in Table S1 in Supporting Information S1.

^a^ Difference between census tracts according to groundwater vulnerability threshold indicated, two‐sample Welch *t*‐test.

^b^ The SVI uses the term “Minority” to refer to “all persons except white, non‐Hispanic.”

^c^ Multi‐unit structures are defined as housing structures with 10 or more units.

^d^ Crowding of an occupied housing unit is defined as having more people than rooms.

^e^ For example, dormitories, residential treatment centers, nursing homes, shelters, military barracks, etc.

## 3 Results and Discussion

### 3.1 Bivariate Analyses

Census tracts with elevated GWV (≥0.001) had a significantly larger proportion of populations below poverty, unemployed, aged 65 or older, and with a reported disability, as well as a larger percentage of mobile homes (Table 1). These areas with elevated GWV also had significantly lower per capita income and population density. Census tracts with non‐elevated GWV (<0.001) had a larger proportion of younger people and individuals in racial/ethnic minority groups, as well as a larger share of multi‐unit structures. Note that there is a low prevalence of minority populations across the study region. Differences in the remaining SVI characteristics were not statistically significant.

### 3.2 Multivariable Analyses

Moderate correlations were observed among the predictors in our data set, alleviating possible issues from multicollinearity (Figure S2 in Supporting Information S1). In the Bayesian spatial multivariable regressions, allvar models with the full set of SVI characteristics exhibited lower DIC (i.e., better performance) than corresponding varsel models that employed variable selection. Population density exhibited the largest statistically significant association, with the odds of elevated GWV decreasing by 18%–20% (e.g., OR = 0.797, 95% CI 0.698, 0.878 for allvar) for every 10 persons/square mile increase in population density. This finding is consistent with the pronounced difference in population density found in the bivariate analyses. It also reflects known patterns of UOG development in the Marcellus and Utica shale regions, where activities tend to be concentrated in less densely populated rural areas. This pattern is in contrast to other regions such as the Barnett shale, where intensive drilling can occur in densely populated urban and suburban neighborhoods (Fry et al., 2015). In Table 2, the odds of a census tract having elevated GWV also decreased 5%–6% (e.g., OR = 0.947, 95% CI 0.896, 0.995 for varsel) for every $1,000 increase in per capita income, indicating that lower income census tracts tended to have higher groundwater vulnerability to contamination. Associations with other SVI characteristics were suggestive but not statistically significant. For example, in the allvar set‐up, elevated GWV was associated with a 12.5% increase (0.4% decrease to 28% increase) for every 1% increase in populations aged 65 or older. In general, the direction of associations (i.e., positive or negative posterior mean) was consistent across allvar and varsel models, except in the case of percent racial/ethnic minorities where a negative association with elevated GWV in allvar changed to a positive association in varsel.

**Table 2:** Odds Ratios (Posterior Means and 95% Credible Intervals) for Associations Between Census Tract‐Level Sociodemographic Characteristics and Elevated Groundwater Vulnerability to Contamination (GWV ≥ 0.001)

Sociodemographic characteristic	Allvar (DIC = 632.1, *p*_D_ = 190.5)	Varsel (DIC = 647.1, *p*_D_ = 141.8)
Below poverty, %	1.014 (0.844, 1.201)	–
Unemployed, %	0.965 (0.748, 1.195)	–
Income per capita, USD	0.934 (0.852, 1.006)	0.947 (0.896, 0.995)^a^
No high school diploma, %	1.023 (0.938, 1.116)	–
Aged 65 or older, %	1.125 (0.996, 1.284)	1.068 (0.986, 1.153)
Aged 17 or younger, %	1.032 (0.882, 1.210)	–
Civilian with a disability, %	0.944 (0.809, 1.087)	–
Single‐parent households, %	1.086 (0.876, 1.360)	–
Minority, %	0.988 (0.709, 1.353)	1.054 (0.871, 1.300)
Aged 5 or older who speaks English “less than well”, %	1.186 (0.553, 2.280)	–
Multi‐unit structures, %	1.033 (0.818, 1.294)	–
Mobile homes, %	1.029 (0.984, 1.081)	1.019 (0.983, 1.058)
Crowding, %	1.042 (0.798, 1.347)	–
No vehicle, %	0.869 (0.683, 1.057)	0.940 (0.845, 1.035)
Group quarters, %	1.039 (0.933, 1.161)	–
Population density, persons/sq mi	0.797 (0.698, 0.878)^a^	0.820 (0.742, 0.894)^a^

*Note*. The OR is the change in odds of elevated GWV for every $1,000 increase in income per capita, for every 10 person/sq mi increase in population density, or for every 1% increase in the remaining % based sociodemographic characteristics.

^a^ Indicates statistical significance; 95% credible interval does not include 1.00.

**Table 3:** Sociodemographic and Residential Characteristics of *n* = 210 Household Survey Respondents in Ohio and West Virginia

Sociodemographic characteristics	*n* (%)	Residential characteristics	*n* (%)
Age		Housing type	
<45	20 (9.5)	Single detached	202 (96.2)
45–64	57 (27.1)	Other	8 (3.8)
≥65	131 (62.4)	Household size	
No response	2 (1.0)	1‐2 people	164 (78.1)
Gender		3‐4 people	34 (16.2)
Male	126 (60.0)	5+ people	11 (5.2)
Female	84 (40.0)	No response	1 (0.5)
Race		Housing ownership	
White	202 (96.2)	Own	204 (97.1)
Other	8 (3.8)	Rent	1 (0.5)
Education		Other	5 (2.4)
No high school diploma	11 (5.2)	Years lived in home	
Post‐high school	143 (68.1)	<1 year	3 (1.4)
Bachelor's	24 (11.4)	1–5 years	25 (11.9)
Post‐bachelor's	32 (15.2)	6–10 years	21 (10.0)
Employment		11–20 years	54 (25.7)
Employed	69 (32.9)	>20 years	107 (51.0)
Retired	121 (57.6)	–	–
Other	20 (9.5)	–	–
Household income		–	–
<$50,000	72 (34.3)	–	–
$50,000–99,999	69 (32.9)	–	–
≥$100,000	47 (22.4)	–	–
No response	22 (10.5)	–	–

*Note*. See Figure 1 for census tracts surveyed.

### 3.3 Sensitivity Analyses

Differences in individual SVI characteristics shown by the bivariate analysis persisted under an elevated GWV threshold of 0.01. Census tracts with higher GWV had a significantly greater proportion of elderly populations and individuals with a reported disability, as well as a lower proportion of younger populations and minorities, lower population density, a lower percentage of multi‐unit structures, and a larger percentage of mobile homes (Table S3 in Supporting Information S1). The detected differences also persisted when limiting the analysis to non‐urban tracts (i.e., those outside the boundaries of census urbanized areas) (Table S4 in Supporting Information S1).

Across all versions of the multivariable spatial regression models, population density and income per capita were consistently associated with a decrease in odds of elevated GWV, while populations aged 65 or older and percentage of mobile homes were consistently associated with increased odds of elevated GWV (Tables S5 and S6 in Supporting Information S1). The magnitude and statistical significance of the associations varied with model version. Predictors appearing only in the allvar models that also exhibited consistent positive associations with elevated GWV were percentage of single parent households, persons with limited English language capacity, and persons living in group quarters, while percent of households with no access to a vehicle exhibited a consistent negative association. Relationships between elevated GWV and the remaining SVI characteristics were mixed, exhibiting either positive or negative associations depending on the spatial regression model set‐up. The majority of the suggestive associations in our study indicate that tracts characterized by higher social vulnerability also generally shoulder higher groundwater vulnerability to contamination from UOG.

Analysis of alternative indicators of social vulnerability, such as Cutter's SVI (Cutter et al., 2003) and the neighborhood deprivation index (Messer et al., 2006), among others, is beyond the scope of the current study. Previous research has demonstrated that various composite indices of social vulnerability are interchangeable due to their high degree of similarity (Deziel, Warren, et al., 2022). However, additional work is needed on the systematic comparison, synthesis, and empirical validation of the underlying sociodemographic characteristics comprising these social vulnerability indices and the types of inequities that they capture.

### 3.4 Findings From the Household Surveys

The majority of survey respondents were aged 65 or older, White, and retired homeowners living in households of 1–2 people (Table 3). Relative to the surrounding census tracts, our sample consists of older residents (62% aged ≥65 years vs. 21% surrounding tract median) with higher educational attainment (5% no high school diploma vs. 13% tract median). Based on the poverty level thresholds defined by the census, 1.9% (4 out of 210 households) were below the poverty level, compared to a 16% median percentage of population below the poverty level in the surrounding census tracts (U.S. Census Bureau, 2021a). Many of our respondents reported residing in their homes for at least 20 years, predating the expansion of UOG development in the Marcellus and Utica Shale.

**Figure 2:**
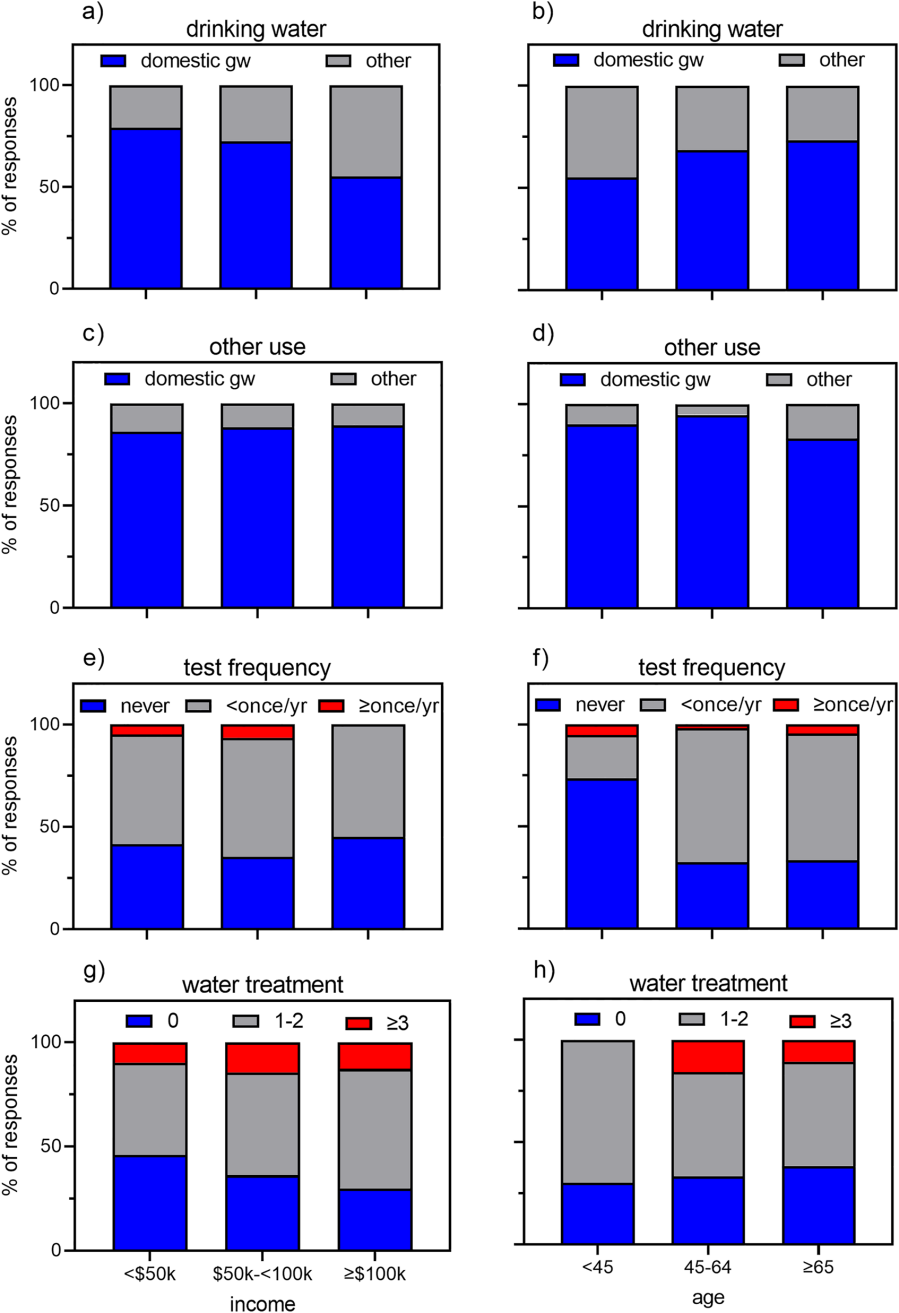
Household water use characteristics by reported income (a, c, e, g) and survey respondent's age (b, d, f, h). Water use characteristics are: source of drinking water (a, b), source of water for other household purposes (c, d), frequency of well water testing (e, f), and number of water treatment devices installed in the home (g, h). In a–d, “domestic gw” includes private groundwater wells and springs while “other” includes bottled water, municipal water, rain barrel, or combinations of domestic groundwater and alternative water sources. In g‐h, treatment devices include water filter, sediment filter, water softener, reverse osmosis, and ultraviolet sterilization.

Information on household water use characteristics is shown in Figure 2. Most respondents reported using domestic groundwater (either a private well or spring) as the sole source of water for drinking (60%) and for other household purposes (75%). The majority of respondents reported testing their well water quality on at least one occasion in the past (63%), but the remainder reported never testing their water (37%). Most respondents also reported installing at least one water treatment device in their homes (64%).

While our survey results cannot be generalized to the entire study region, we can still derive notable insights from these data. Household income was significantly associated with the source of drinking water (Fisher's exact test *p*‐value = 0.02155), with higher income households more likely to report having access to alternative sources such as bottled and municipal water. Households in the lowest income bracket were also the most likely to report having no water treatment devices installed. Respondent age was significantly associated with the frequency of well water testing (Fisher's exact test *p*‐value = 0.006577), with older respondents more likely to report testing their water at least once per year. These results suggest that sociodemographic characteristics indeed capture a household's ability to assess and mitigate exposures to contaminated waters and, thus, disparities in the former can reflect disparities in the latter.

### 3.5 Implications

The physically based assessment of groundwater vulnerability to contamination provides a novel approach for characterizing risks posed by UOG specific to drinking water exposures, which cannot be disaggregated from other exposure pathways (e.g., air) using distance‐based proxies alone. The ability to identify specific drinking water contamination risks is critical for communities that heavily depend on groundwater for daily use, as such risks can be potentially mitigated by household access to alternative water sources, treatment systems, and frequent well water testing. Our assessment of groundwater vulnerability to contamination and sociodemographic characteristics suggests potential environmental injustice related to a disproportionate burden of UOG risks on socially vulnerable communities. We found disproportionately elevated GWV among lower income populations, which can constitute an environmental injustice as these populations may have reduced capacity to mitigate the exposures and impacts of UOG due to lower access to healthcare, lack of alternative drinking water sources, or cumulative burdens from a multiplicity of environmental hazards (Khanassov et al., 2016; Wrenn et al., 2016). We also saw increased GWV among communities with older populations, which may pose public health challenges given the physiologic susceptibilities of the elderly to environmental stressors, including degraded drinking water quality (Beaudeau et al., 2014; Geller & Zenick, 2005; Hong, 2013). Indeed, a recent national scale study found statistically significant increases in all‐cause mortality among elderly populations living near UOG sites, highlighting the urgency of research focused on these vulnerable populations (Li et al., 2022). Additional empirical work is needed to elucidate the social processes that drive these observed associations, for instance, whether the association between elevated GWV and greater elderly populations is the result of aging in place (i.e., Are elderly residents more likely to own and lease their mineral rights?) or migration (i.e., Are elderly people moving into retirement destinations that host intensive UOG activities?) (Smith & Trevelyan, 2019). Our household survey results support the former, as our respondents reported living in their homes prior to the onset of UOG development in their communities. However, our survey did not directly ask respondents if they had leased their mineral rights to UOG companies and, if so, the drivers of those decisions. Future interdisciplinary research should probe these processes further.

Our regional tract‐level analyses also suggested associations between elevated GWV and higher proportions of mobile homes and persons living in group quarters. Populations in these settings have a lower likelihood of owning land or mineral rights, and thus are potentially exposed to contamination risks without accruing any direct economic benefits from UOG development. Indeed, scenarios where the largest benefits of UOG go to those who bear little to none of the environmental and public health risks have been identified by previous research in the benefit‐sharing environmental justice literature (Clough & Bell, 2016; Fry et al., 2015; Hardy & Kelsey, 2015; Lieberman‐Cribbin et al., 2022). Residents who do not own land or mineral rights may also face procedural injustice as they have limited mechanisms to influence and consent to leasing and drilling decisions, and may lack recognition as legitimate stakeholders to participate in policymaking processes (Baka et al., 2019; Jalbert et al., 2019; Whitton et al., 2017).

## 4 Conclusions

This work contributes to the mounting body of research showing disparities in the distribution of impacts and risks from UOG development. Our physically based source‐pathway‐receptor vulnerability modeling framework provides a novel, spatially explicit approach for evaluating groundwater contamination risks from UOG development. This mechanistic framework characterizes risk pathways specific to drinking water pollution, whereas previous distance‐based proxies only describe aggregate risks. The physically based vulnerability analysis thus provides a more focused lens with which to examine questions of environmental justice. Regional estimates of groundwater vulnerability to contamination from UOG activities were consistently elevated in communities exhibiting certain social vulnerability characteristics– lower income per capita, larger proportions of elderly populations, and larger percentages of mobile homes– suggesting the potential occurrence of distributive environmental injustice. Our household surveys provide empirical support documenting relationships between sociodemographic characteristics and a household's capacity to address domestic groundwater contamination. Additional research is needed on the social processes driving these associations and on the degree to which the observed sociodemographic disparities in groundwater vulnerability translate to differences in public health endpoints such as disease and mortality, as well as on the other dimensions of environmental justice.

## Conflict of Interest

The authors declare no conflicts of interest relevant to this study.

## Supporting information

Supporting Information S1

## Data Availability

Data supporting the findings of this study are available on Zenodo (Soriano et al., 2023, https://doi.org/10.5281/zenodo.7641586). Individual survey responses are protected by IRB confidentiality protocols. All statistical analyses were performed using the open source R software environment.
