# Supplementary material for: Social Vulnerability and Groundwater Vulnerability to Contamination From Unconventional Hydrocarbon Extraction in the Appalachian Basin
Source: Geohealth. 2023 Apr 13;7(4):e2022GH000758. doi: 10.1029/2022GH000758 (PMC10100439; doi:10.1029/2022GH000758)
Supplement: Supplementary file 1 — Supporting Information S1 [file GH2-7-e2022GH000758-s001.docx]

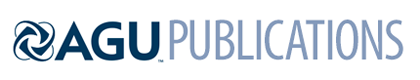


*GeoHealth*

Supporting Information for

**Social vulnerability and groundwater vulnerability to contamination from unconventional hydrocarbon extraction in the Appalachian Basin**

Mario A. Soriano Jr.,^1,2^ Joshua L. Warren,^3^ Cassandra J. Clark,^4^ Nicholaus P. Johnson,^4^ Helen G. Siegel,^1^ Nicole C. Deziel,^4^ and James E. Saiers^1^

^1^ School of the Environment, Yale University, New Haven, CT 06511, United States of America

^2^ Integrated GroundWater Modeling Center, High Meadows Environmental Institute, Princeton University, Princeton, NJ 08544, United States of America

^3^ Department of Biostatistics, School of Public Health, Yale University, New Haven, CT 06510, United States of America

^4^ Department of Environmental Health Sciences, School of Public Health, Yale University, New Haven, CT 06510, United States of America

*Contents:*

**Figure S1.** Map of groundwater vulnerability to contamination.

**Table S1.** Sociodemographic characteristics used in the CDC’s Social Vulnerability Index.

**Table S2.** Weakly informative prior distributions for the spatial regression model parameters.

**Table S3.** Sensitivity analysis of bivariate results using GWV threshold = 0.01.

**Table S4.** Sensitivity analysis of bivariate results using non-urban census tracts.

**Figure S2.** Correlation coefficient between SVI characteristics.

**Table S5.** Sensitivity analysis of multivariable regression results using GWV threshold = 0.01.

**Table S6.** Sensitivity analysis of multivariable regression results using non-urban census tracts.

**Text S1.** Questions from the homeowner survey.


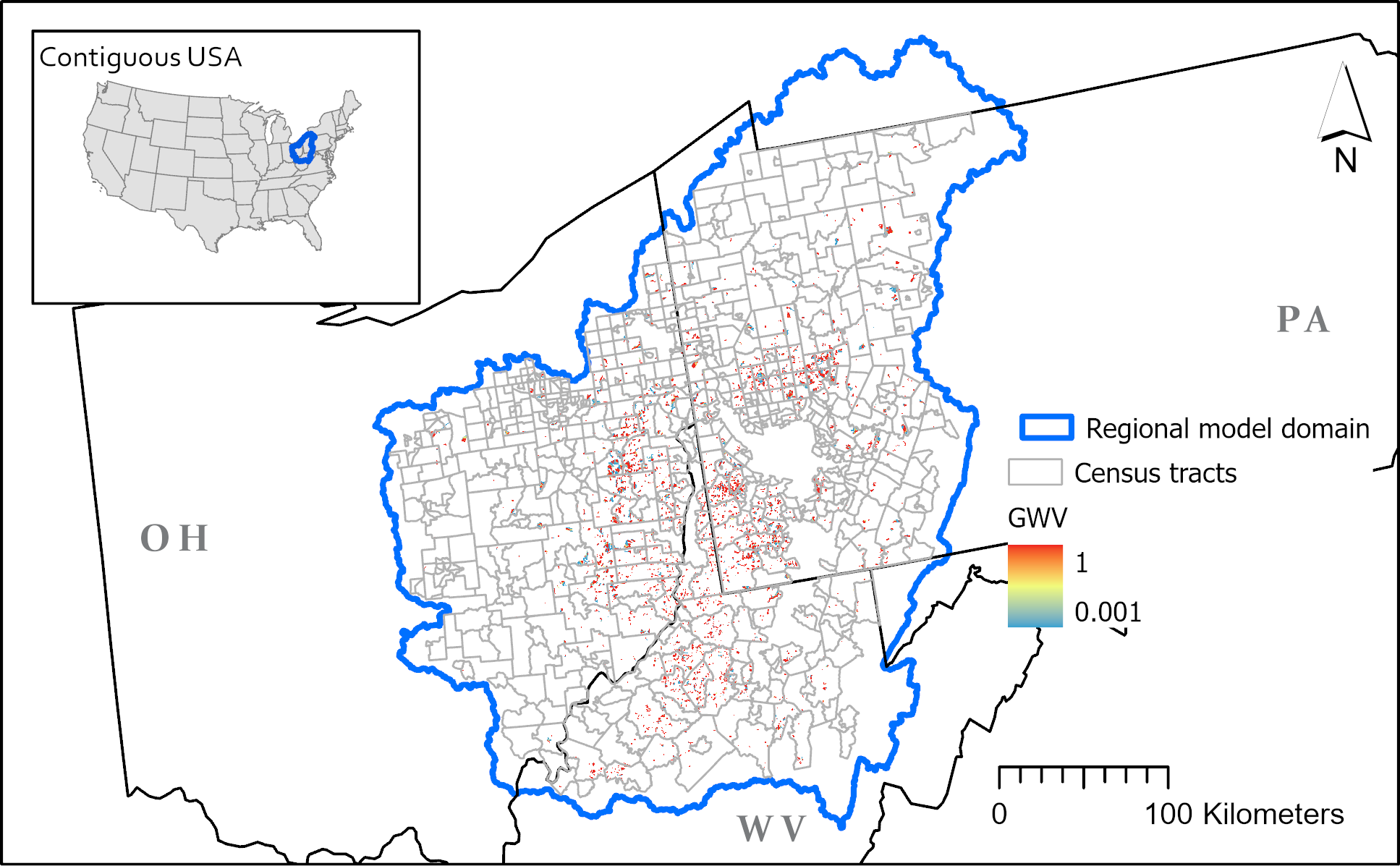


**Figure S1.** Groundwater vulnerability to contamination (GWV) in the regional model domain. Census tracts shown include those where the proportion of the population dependent on domestic groundwater supplies was ≥10% (*n* = 647) according to estimates from Murray et al.^1^

**Table S1.** Sociodemographic characteristics used in the CDC’s Social Vulnerability Index.^2^

| *Variable* | *Definition* | *Source* |
| --- | --- | --- |
| EP_POV | % population below poverty level | SVI 2018 shapefile |
| EP_UNEMP | % unemployed population | SVI 2018 shapefile |
| EP_PCI | per capita income ($) | SVI 2018 shapefile |
| EP_NOHSDP | % population with no high school diploma | SVI 2018 shapefile |
| EP_AGE65 | % population 65 y/o or older | SVI 2018 shapefile |
| EP_AGE17 | % population 17 y/o or younger | SVI 2018 shapefile |
| EP_DISABL | % population with disability | SVI 2018 shapefile |
| EP_SNGPNT | % single parent households out of total households | SVI 2018 shapefile |
| EP_MINRTY | % minority populations (all persons except white, non-Hispanic) | SVI 2018 shapefile |
| EP_LIMENG | % population with limited English language capacity (native language is not English; speak English “not well” or “not at all) | SVI 2018 shapefile |
| EP_MUNIT | % multiunit structures (housing structures with 10 or more units) out of total housing units | SVI 2018 shapefile |
| EP_MOBILE | % mobile homes out of total housing units | SVI 2018 shapefile |
| EP_CROWD | % housing units with more people than rooms out of total housing units | SVI 2018 shapefile |
| EP_NOVEH | % households with no vehicle out of total households | SVI 2018 shapefile |
| EP_GROUPQ | % population living in group quarters (e.g., nursing homes, college dormitories, military barracks, shelters, correctional facilities, mental hospitals) | SVI 2018 shapefile |
| EP_POPDENS | population density (persons per square mile) | calculated as E_TOTPOP/AREA_SQMI (both from SVI 2018 shapefile) |

**Table S2.** Weakly informative prior distributions for the spatial regression model parameters.

| Parameter | Prior Distribution | Description |
| --- | --- | --- |
| β | Normal (0,10000) | unknown regression parameters describing associations between outcome and predictor |
| τ^2^ | Inverse Gamma (10,10) | variability in the random effect terms |
| ρ | Uniform (0,1) | spatial dependence term |

**Table S3.** Sensitivity analysis of bivariate results using GWV threshold = 0.01. Sociodemographic characteristics of tracts categorized by groundwater vulnerability to contamination. Data are shown as medians (25th – 75th percentiles).

| Sociodemographic characteristic | tracts with ≥ 10% gw dependent population (*n* = 647) | | |
| --- | --- | --- | --- |
|  | GWV ≥ 0.01 (*n* = 256) | GWV < 0.01 (*n* = 391) | *p*-value*^a^* |
| *SVI Theme: Socioeconomic Status* | | | |
| Below poverty, % | 10.5 (6.9 - 14.6) | 10.3 (6.0 - 14.6) | 0.677 |
| Unemployed, % | 4.9 (3.5 - 6.4) | 4.6 (3.1 - 6.4) | 0.531 |
| Income per capita, USD | 28058 (25164 - 32130) | 27389 (24329 - 32558) | 0.076 |
| No high school diploma, % | 8.7 (6.4 - 12.4) | 8.8 (6.0 - 12.4) | 0.366 |
|  |  |  |  |
| *SVI Theme: Household Composition & Disability* | | | |
| Aged 65 or older, % | 20.4 (18.5 - 22.6) | 19.3 (16.4 - 21.8) | <0.001 |
| Aged 17 or younger, % | 19.6 (17.7 - 21.7) | 20.9 (18.4 - 23.6) | <0.0001 |
| Civilian with a disability, % | 15.7 (13.1 - 18.6) | 15 (11.8 - 18.0) | 0.011 |
| Single-parent households, % | 6.1 (4.3 - 8.3) | 6.3 (4.1 - 8.5) | 0.099 |
|  |  |  |  |
| *SVI Theme: Minority Status & Language* | | | |
| Minority*^b^*, % | 3.1 (1.9 - 4.8) | 3.8 (2.2 - 6.6) | 0.024 |
| Aged 5 or older who speaks English "less than well", % | 0.0 (0.0 - 0.3) | 0.1 (0.0 - 0.5) | 0.013 |
|  |  |  |  |
| *SVI Theme: Housing Type & Transportation* | | | |
| Multi-unit structures*^c^*, % | 0.4 (0 - 2.1) | 0.9 (0.0 - 3.2) | 0.045 |
| Mobile homes, % | 12.4 (6.7 - 18.3) | 9.1 (2.9 - 15.5) | <0.001 |
| Crowding*^d^*, % | 0.7 (0.1 - 1.6) | 0.8 (0.0 - 1.6) | 0.135 |
| No vehicle, % | 4.3 (2.9 - 6.2) | 4.6 (2.7 - 7.1) | 0.031 |
| Group quarters*^e^*, % | 0.3 (0.0 - 2) | 0.2 (0.0 - 1.7) | 0.357 |
|  |  |  |  |
| Population density, persons/sq mi | 112.9 (53.9 - 194.1) | 158.3 (75.0 - 474.3) | <0.0001 |
| *^a^* Difference between census tracts according to groundwater vulnerability threshold indicated, two-sample Welch *t*-test | | | |
| *^b^* The SVI uses the term “Minority” to refer to “all persons except white, non-Hispanic” | | | |
| *^c^* Multi-unit structures are defined as housing structures with 10 or more units | | | |
| *^d^* Crowding of an occupied housing unit is defined as having more people than rooms | | | |
| *^e^* e.g., dormitories, residential treatment centers, nursing homes, shelters, military barracks, etc. | | | |

**Table S4.** Sensitivity analysis of bivariate results using non-urban census tracts. Sociodemographic characteristics of tracts categorized by groundwater vulnerability to contamination. Data are shown as medians (25^th^ – 75^th^ percentiles).

| Sociodemographic characteristic | non-urban tracts (*n* = 646) | | | non-urban tracts (*n* = 646) | | |
| --- | --- | --- | --- | --- | --- | --- |
|  | GWV ≥ 0.001 (*n* = 383) | GWV < 0.001 (*n* = 263) | *p*-value*^a^* | GWV ≥ 0.01 (*n* = 275) | GWV < 0.01 (*n* = 371) | *p*-value*^a^* |
| *SVI Theme: Socioeconomic Status* | | | | | | |
| Below poverty, % | 11.5 (8.0 - 16.0) | 10.7 (7.2 - 15.5) | 0.807 | 11.3 (7.9 - 15.9) | 11.1 (7.6 - 15.7) | 0.863 |
| Unemployed, % | 5.0 (3.6 - 7.0) | 4.6 (3.0 - 6.5) | 0.046 | 5.0 (3.6 - 6.8) | 4.8 (3.1 - 6.8) | 0.429 |
| Income per capita, USD | 26848 (24181 - 30244) | 27397 (23907 - 32314) | 0.013 | 27470 (24672 - 31176) | 26774 (23879 - 31271) | 0.444 |
| No high school diploma, % | 9.7 (7.0 - 13.3) | 8.8 (6.2 - 13.9) | 0.86 | 9.3 (6.7 - 12.9) | 9.4 (6.7 - 13.2) | 0.148 |
| *SVI Theme: Household Composition & Disability* | | | | | | |
| Aged 65 or older, % | 20.2 (17.9 - 22.4) | 19.5 (16.3 - 22.0) | 0.015 | 20.3 (18.4 - 22.5) | 19.5 (16.5 - 22.0) | 0.022 |
| Aged 17 or younger, % | 19.7 (17.9 - 21.8) | 20.2 (18.1 - 23.4) | 0.024 | 19.6 (17.8 - 21.7) | 20.3 (18.1 - 23.1) | 0.003 |
| Civilian with a disability, % | 16.3 (13.8 - 19.4) | 15.4 (12.4 - 18.3) | 0.004 | 16.3 (13.9 - 19.4) | 15.7 (12.8 - 18.5) | 0.026 |
| Single-parent households, % | 6.3 (4.5 - 8.5) | 6.2 (4.2 - 8.5) | 0.432 | 6.2 (4.4 - 8.6) | 6.3 (4.4 - 8.4) | 0.328 |
| *SVI Theme: Minority Status & Language* | | | | | | |
| Minority, % | 3.0 (1.9 - 4.5) | 4.0 (2.4 - 6.5) | 0.003 | 3 (1.9 - 4.6) | 3.6 (2.2 - 5.7) | 0.022 |
| Aged 5 or older who speaks English "less than well", % | 0.0 (0.0 - 0.3) | 0.1 (0.0 - 0.5) | 0.178 | 0.0 (0.0 - 0.3) | 0.0 (0.0 - 0.4) | 0.039 |
| *SVI Theme: Housing Type & Transportation* | | | | | | |
| Multi-unit structures, % | 0.4 (0.0 - 2.0) | 1.1 (0.0 - 3.8) | 0.001 | 0.3 (0.0 - 2.0) | 0.7 (0.0 - 3.1) | 0.047 |
| Mobile homes, % | 13.1 (7.7 - 18.1) | 9.5 (3.5 - 15.9) | <0.0001 | 12.7 (7.3 - 18.4) | 10.5 (5.1 - 16.4) | 0.019 |
| Crowding, % | 0.9 (0.2 - 1.7) | 0.8 (0.2 – 2.0) | 0.169 | 0.8 (0.1 - 1.7) | 0.9 (0.2 - 2.0) | 0.015 |
| No vehicle, % | 4.7 (3.1 - 7.1) | 5 (3.2 - 7.9) | 0.063 | 4.7 (3.1 – 7.0) | 4.9 (3.1 - 7.7) | 0.048 |
| Group quarters, % | 0.2 (0.0 - 1.6) | 0.2 (0.0 - 1.7) | 0.397 | 0.3 (0.0 - 1.8) | 0.2 (0.0 - 1.7) | 0.839 |
| Population density, persons/sq mi | 101.0 (53.3 - 178.1) | 185.2 (96.6 - 375.1) | <0.0001 | 112.7 (56.2 - 189.5) | 139.4 (71.0 - 307.7) | 0.009 |
| *^a^* Difference between census tracts according to groundwater vulnerability threshold indicated, two-sample Welch *t*-test | | | | | | |

a)


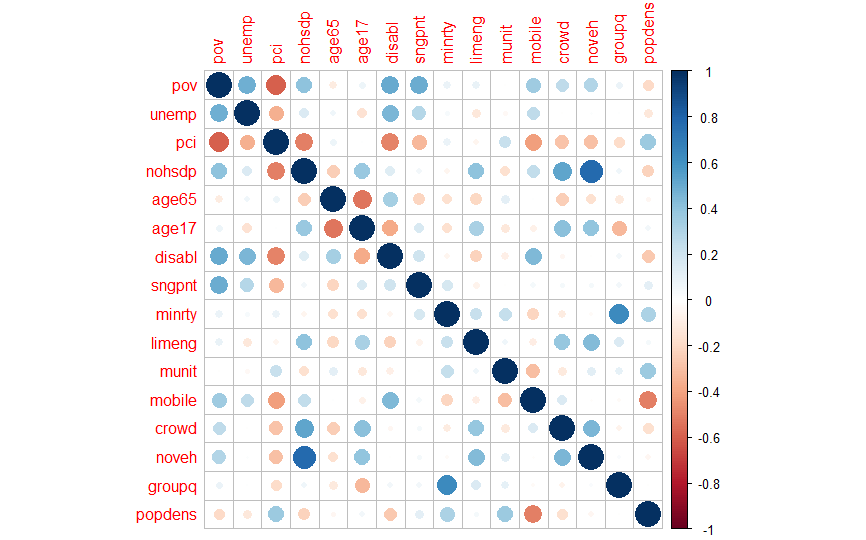


b)


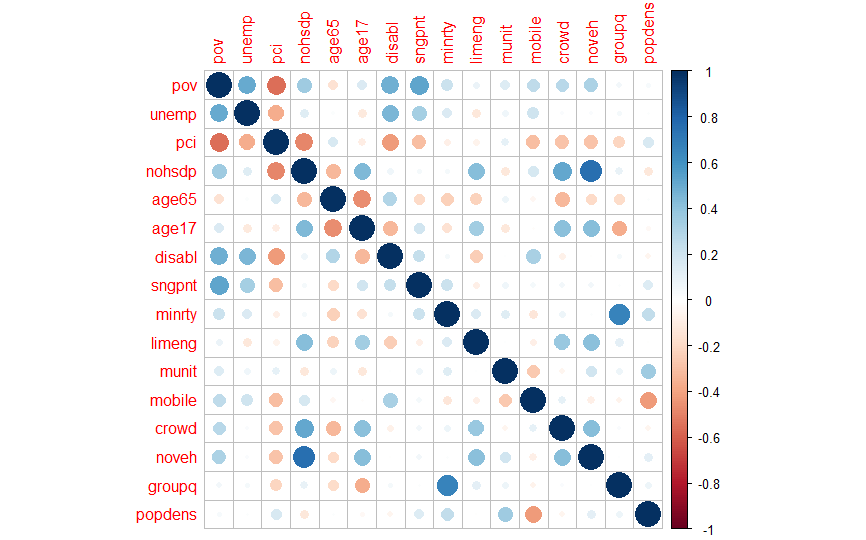


**Figure S2.** Correlation coefficient between SVI characteristics for a) census tracts with groundwater dependent populations >10% of total tract population,^1^ and b) census tracts outside census urbanized areas (i.e., non-urban tracts).^3^

**Table S5.** Sensitivity analysis of multivariable regression results using GWV threshold = 0.01. Odds ratios (posterior means and 95% credible intervals) for associations between census tract-level sociodemographic characteristics and elevated groundwater vulnerability to contamination (GWV ≥ 0.01). The OR is the change in odds of elevated GWV for every $1000 increase in income per capita, for every 10 person/sq mi increase in population density, or for every 1% increase in the remaining % based sociodemographic characteristics.

| Sociodemographic characteristic | allvar | varsel |
| --- | --- | --- |
|  | (DIC = 630.8, p_D_ = 198.4) | (DIC = 656.5, p_D_ = 136.1) |
| Below poverty, % | 1.089 (0.912, 1.303) |  |
| Unemployed, % | 0.799 (0.573, 0.996)*^a^* |  |
| Income per capita, USD | 0.946 (0.850, 1.024) | 0.968 (0.918, 1.014) |
| No high school diploma, % | 1.002 (0.913, 1.101) |  |
| Aged 65 or older, % | 1.152 (1.020, 1.345)*^a^* | 1.081 (0.992, 1.181) |
| Aged 17 or younger, % | 1.024 (0.865, 1.211) | 0.962 (0.869, 1.065) |
| Civilian with a disability, % | 0.984 (0.848, 1.143) |  |
| Single-parent households, % | 1.035 (0.820, 1.304) |  |
| Minority, % | 0.794 (0.514, 1.098) | 0.983 (0.804, 1.182) |
| Aged 5 or older who speaks English "less than well", % | 1.013 (0.432, 1.994) |  |
| Multi-unit structures, % | 0.969 (0.734, 1.233) |  |
| Mobile homes, % | 1.016 (0.969, 1.070) | 1.010 (0.975, 1.046) |
| Crowding, % | 0.976 (0.722, 1.302) |  |
| No vehicle, % | 0.889 (0.675, 1.095) |  |
| Group quarters, % | 1.124 (1.002, 1.299)*^a^* |  |
| Population density, persons/sq mi | 0.853 (0.713, 0.955)*^a^* | 0.868 (0.781, 0.937) *^a^* |
| *^a^* Indicates statistical significance; 95% credible interval does not include 1.00 | |  |

**Table S6.** Sensitivity analysis of multivariable regression results using non-urban census tracts. Odds ratios (posterior means and 95% credible intervals) for associations between census tract-level sociodemographic characteristics and elevated groundwater vulnerability to contamination. The OR is the change in odds of elevated GWV for every $1000 increase in income per capita, for every 10 person/sq mi increase in population density, or for every 1% increase in the remaining % based sociodemographic characteristics.

| Sociodemographic characteristic | elevated GWV threshold = 0.001 | | elevated GWV threshold = 0.01 | |
| --- | --- | --- | --- | --- |
|  | allvar | varsel | allvar | varsel |
|  | (DIC = 611.3, p_D_ = 199.8) | (DIC = 635.0, p_D_ = 154.6) | (DIC = 632.2, p_D_ = 183.8) | (DIC = 643.9, p_D_ = 148.5) |
| Below poverty, % | 0.978 (0.826, 1.146) | 0.979 (0.873, 1.087) | 1.046 (0.895, 1.217) |  |
| Unemployed, % | 1.039 (0.824, 1.293) |  | 0.913 (0.733, 1.106) |  |
| Income per capita, USD | 0.887 (0.781, 0.981)*^a^* | 0.904 (0.828, 0.974)*^a^* | 0.909 (0.796, 1.001) | 0.914 (0.844, 0.977)*^a^* |
| No high school diploma, % | 1.003 (0.912, 1.098) |  | 0.983 (0.898, 1.066) |  |
| Aged 65 or older, % | 1.064 (0.943, 1.208) | 1.047 (0.953, 1.151) | 1.061 (0.948, 1.190) | 1.040 (0.950, 1.140) |
| Aged 17 or younger, % | 1.046 (0.875, 1.246) | 1.012 (0.908, 1.129) | 1.069 (0.911, 1.257) | 0.998 (0.891, 1.114) |
| Civilian with a disability, % | 1.023 (0.868, 1.203) |  | 1.056 (0.912, 1.217) |  |
| Single-parent households, % | 1.085 (0.861, 1.384) |  | 1.009 (0.812, 1.244) |  |
| Minority, % | 0.815 (0.569, 1.061) | 0.919 (0.770, 1.080) | 0.753 (0.546, 1.007) | 0.922 (0.770, 1.080) |
| Aged 5 or older who speaks English "less than well", % | 1.802 (0.785, 3.916) |  | 1.219 (0.550, 2.332) |  |
| Multi-unit structures, % | 0.918 (0.614, 1.272) |  | 0.921 (0.652, 1.240) |  |
| Mobile homes, % | 1.029 (0.981, 1.085) | 1.028 (0.990, 1.072) | 1.010 (0.965, 1.059) | 1.011 (0.974, 1.049) |
| Crowding, % | 0.982 (0.751, 1.253) |  | 0.936 (0.716, 1.185) | 0.926 (0.751, 1.125) |
| No vehicle, % | 0.898 (0.699, 1.100) |  | 0.939 (0.760, 1.145) |  |
| Group quarters, % | 1.035 (0.942, 1.147) |  | 1.093 (0.994, 1.210) |  |
| Population density, persons/sq mi | 0.734 (0.552, 0.908)*^a^* | 0.755 (0.636, 0.864)*^a^* | 0.852 (0.687, 1.020) | 0.830 (0.729, 0.937)*^a^* |
| *^a^* Indicates statistical significance; 95% credible interval does not include 1.00 | |  |  |  |

**Text S1.** Questions from the homeowner survey.

Q1. How many years have you lived in your home?

Q2. Do you own or rent your home?

Q3. How many people currently live in your home?

Q4. Which of the following best describes the home?

1 – A one-family house detached from any other house

2 – A one-family house attached to one or more houses (for example Row house/Town house)

3 – A building with 10 or fewer apartments

4 – A building with more than 10 apartments

5 – Mobile home, Boat, RV, van, or other

Q5. What is your date of birth (MM/DD/YYYY)?

Q6. What is your gender?

Q7. In which of these groups would you place yourself?

1 – White

2 – Black or African American

3 – American Indian/Alaska Native

4 – Asian

5 – Native Hawaiian or Other Pacific Islander

6 – Other race

7 – More than one race

Q8. What is the highest year of school or college that you completed?

Q9. What is your primary employment status?

1 – Employed full time

2 – Employed part time

3 – Self-employed

4 – Homemaker

5 – Student

6 – Retired

7 – Unemployed

8 – Unable to work

9 – Other. Please specify:

Q10. What best describes your household income in the past year?

1 – < $25,000

2 – $25,000 - $49,999

3 – $50,000 - $74,999

4 – $75,000 - $99,999

5 – $100,000 - $149,000

6 – $150,000 - $199,999

7 – $200,000 or more

B – No answer

M – Don’t know

Q11. What is the main source of drinking water for members of your household?

1 – Private well

2 – Spring

3 – Municipal (city) water piped to the home

4 – Bottled water

5 – Public tap

6 – Rain barrel

7 – Creek or river

8 – Other. Please specify:

**Text S1 continued.**

Q12. What is the main source of water used by your household for purposes OTHER than drinking, such as cooking, showering, hand-washing, and laundry?

1 – Private well

2 – Spring

3 – Municipal (city) water piped to the home

4 – Bottled water

5 – Public tap

6 – Rain barrel

7 – Creek or river

8 – Other. Please specify:

Q13. Do you currently use any of the following to treat your drinking water?

1 – Under-the-sink, faucet-mounted, refrigerator dispenser, or countertop water filter

2 – An in-line water treatment system that uses reverse osmosis

3 – An in-line water treatment system that uses UV sterilization

4 – An in-line water treatment system that uses a water softener system

5 – An in-line water treatment system that uses sediment filter

6 – Any other water treatment system. Please specify:

Q14. How frequently do you have the water from your well tested?

0 – Never

1 – Less than once per year

2 – Once per year

3 – More than once per year

**References**

1. Murray, A.; Hall, A.; Weaver, J.; Kremer, F., Methods for Estimating Locations of Housing Units Served by Private Domestic Wells in the United States Applied to 2010. *JAWRA Journal of the American Water Resources Association* **2021,** *57*, (5), 828-843.

2. Centers for Disease Control and Prevention/ Agency for Toxic Substances and Disease Registry/ Geospatial Research Analysis and Services Program CDC/ATSDR Social Vulnerability Index 2018 Database US. https://www.atsdr.cdc.gov/placeandhealth/svi/data_documentation_download.html (02/01/2022).

3. U.S. Census Bureau TIGER/Line Shapefile, 2018, 2010 nation, U.S., 2010 Census Urban Area National. https://catalog.data.gov/dataset/tiger-line-shapefile-2018-2010-nation-u-s-2010-census-urban-area-national (02/01/2022),
